# The impact of nonlinear exposure-risk relationships on seasonal time-series data: modelling Danish neonatal birth anthropometric data

**DOI:** 10.1186/1471-2288-7-45

**Published:** 2007-10-15

**Authors:** John McGrath, Adrian Barnett, Darryl Eyles, Thomas Burne, Carsten B Pedersen, Preben Bo Mortensen

**Affiliations:** 1 Queensland Centre for Mental Health Research, The Park Centre for Mental Health, Wacol, Q4076, Australia; 2 Department of Psychiatry, University of Queensland, St Lucia, Q4076 Australia; 3 School of Population Health, University of Queensland, Herston, Q4006, Australia; 4 National Centre for Register-based Research, Taasingegade 1, University of Aarhus 8000 Aarhus C, Denmark

## Abstract

**Background:**

Birth weight and length have seasonal fluctuations. Previous analyses of birth weight by latitude effects identified seemingly contradictory results, showing both 6 and 12 monthly periodicities in weight. The aims of this paper are twofold: (a) to explore seasonal patterns in a large, Danish Medical Birth Register, and (b) to explore models based on seasonal exposures and a non-linear exposure-risk relationship.

**Methods:**

Birth weight and birth lengths on over 1.5 million Danish singleton, live births were examined for seasonality. We modelled seasonal patterns based on linear, U- and J-shaped exposure-risk relationships. We then added an extra layer of complexity by modelling weighted population-based exposure patterns.

**Results:**

The Danish data showed clear seasonal fluctuations for both birth weight and birth length. A bimodal model best fits the data, however the amplitude of the 6 and 12 month peaks changed over time. In the modelling exercises, U- and J-shaped exposure-risk relationships generate time series with both 6 and 12 month periodicities. Changing the weightings of the population exposure risks result in unexpected properties. A J-shaped exposure-risk relationship with a diminishing population exposure over time fitted the observed seasonal pattern in the Danish birth weight data.

**Conclusion:**

In keeping with many other studies, Danish birth anthropometric data show complex and shifting seasonal patterns. We speculate that annual periodicities with non-linear exposure-risk models may underlie these findings. Understanding the nature of seasonal fluctuations can help generate candidate exposures.

## Background

Birth weight has long been acknowledged as an important measure of neonatal health [1]. In addition to providing insights into prenatal development, this variable is known to be associated with a wide range of important cognitive, behavioural and health outcomes in infancy, childhood and adulthood. While there is widespread agreement that birth weight shows seasonal fluctuations [2,3], there is a lack of between-location agreement about features such as: (a) the timing (or phase) of maximum weight, and (b) the presence of a single peak (unimodal, 12 month periodicity) or two peaks (bimodal, 6 and 12 month periodicities). With respect to the phase of peak birth weight, some populations had regular, sine wave-like 12 month periodicities, with peak birth weight in spring [4], while other studies found no apparent seasonal fluctuation [5], or a summer peak for birth weight [6,7]. With respect to periodicity, several studies have reported bimodalilty, with 6 and 12 month signals [4,7,8].

Apart from seasonal fluctuations in birth weight, birth length also has an annual periodicity [4,9,10]. Using a population sample drawn from the Danish Medical Birth Register, Wohlfahrt and colleagues reported annual fluctuations in birth length [11], with peak birth length in April (Spring), and a second smaller peak in October. Finally, the frequency of premature birth, a variable that can influence both birth weight and birth length, has been shown to have both six and twelve month seasonality in different sites [12].

Our ability to analyse seasonality in time series data has improved substantially in recent decades, with more robust and flexible statistical models now available to capture the complexities of the data [13]. Recently, we applied these methods to examine the interaction between seasonal fluctuations in birth weight and latitude in four regions in Australia [14]. In keeping with a previous study from Japan [8], we identified: (a) a signal with a 6 month periodicity in addition to the expected 12 month signal, and (b) the amplitude of the two signals varied over latitude (in particular, the amplitude of the smaller 6 month signal increased further away from the equator).

The interpretation of these data has been a challenge to the research community. The presence of both 6 month and 12 month signals have lead to the understandable conclusion that at least two different 'source generators' must be driving these signals. To further complicate matters there are two scenarios, either: (a) two source generators of different amplitudes, each with 12 month periodicities, but phase shifted by 6 months (summing to produce both 6 and 12 month periodicities), or (b) one source generator with a signal of 12 months and a second source generator with a nested harmonic with a 6 month period. It is difficult to identify biologically plausible candidate exposures that would explain such patterns.

To date, seasonality models have been based on the unspoken assumption that the Exposure-Risk Relationship (ERR) is linear. In other words, as the seasonally-fluctuating exposure changes over the year, the biological variable of interest responds in a linear fashion. However, J-shaped or U-shaped ERRs are relatively common in perinatal epidemiology and related research [15-22]. It seems that important developmental systems are able to adjust to a wide range of inputs (i.e. the pathways are 'buffered'), and adverse outcomes only emerge above and/or below certain critical thresholds. If, for example, birth weight was compromised by both high and low ambient temperatures during the last trimester, seasonal analyses from sites that had a wide range of temperature would be expected to have a six month periodicity (drop in birth weight in both summer and winter). We predict that exposures with a 12 month periodicity, but an underlying nonlinear (J-shaped or U-shaped) ERR, will result in time series which generate both 6 and 12 month periodicities.

A second unspoken assumption of seasonal analyses to date is that the entire population experiences the full range of exposure. There is reason to believe that some portions of the population may only experience parts of the exposure range. For example, some portion of the population may be 'partitioned' to one segment of the J- or U-shaped ERR. Using our previous hypothetical relationship between ambient temperature during the third trimester and birth weight, if sites that were exposed to only one temperature extreme (i.e. temperature only ranged between optimal and high, or optimal and low), then seasonal analyses from such sites would show a 12 monthly periodicity. By partitioning different fractions of the populations within different subregions of the J- or U-shaped ERR (e.g. the linear upper segment versus the nonlinear lower segment), we predict that the resultant summed time series of the dependent variable will be altered.

We are not aware that these two assumptions have been previously explored in seasonality research. Because the models involve a number of stages and are perhaps not intuitive, modelling exercises can assist in demonstrating their properties. We aim to provide alternative models that could help explain some of the complexities of seasonal fluctuations in birth weight and birth length data, as well as explain some of the differences in published results.

## Methods

In the first part of the results we will present the data from the Danish Medical Birth register [23], and in the second part we will present various models based on linear versus nonlinear exposure-risk relationships.

### Danish perinatal data

Data were derived from the Danish National Centre for Register-based Research. We examined birth weight and birth length in all live-born, singletons with gestations of at least 37 weeks born between 1973 and 2002 (includes births in 2002). In order to reduce the possible influence of migrant-status on birth anthropometry, we included only births where both the mother and father had been born in Denmark.

We applied similar techniques to those used in the study of Australian perinatal registers [14]. Records of individual birth weights were summarised into a time series of monthly means. The trend and seasonality in these means were then extracted using the 'combined' method described in Barnett and Dobson [13]. This method splits the time series into three parts: long-term trend, season(s) and residual noise. The trend is fitted using a cubic spline, and hence is possibly non-linear. Season (at time *t*) is modelled using a sinusoidal form:

Season [time = *t*] = amplitude × cosine (period × *t *+ phase),

where the phase controls the location of the seasonal peak, and the amplitude controls its size. For the analyses presented here the period was fixed to follow a 6 or 12 month cycle. It is possible to specify multiple seasonal components with different periods. An important feature of this method is that it is a dynamic model, and hence the estimated seasonal pattern is allowed to change over time. Methods based on averaging the entire dataset by month [7,24] assume that the amplitude and phase of the seasonal pattern remains constant over time (known as a stationary model). This dynamism of the model is created by allowing the amplitude and phase to vary over time, hence the above equation becomes

Season [time = *t*] = amplitude [time = *t*] × cosine (period × *t *+ phase [time = *t*]).

This is known as a non-stationary model, because season is not necessarily fixed in time. The non-stationarity in the amplitude and phase are modelled using an autoregressive structure, so that the seasonal estimate at time *t *is dependent on the estimate at time *t*-1. Using this structure any change in seasonality (e.g. a decreasing amplitude over time) is assumed to occur gradually.

Because the model is dynamic and involves the non-linear cosine function it cannot be fitted using standard methods, such as maximum likelihood. Instead the method uses Markov chain Monte Carlo (MCMC) to estimate the joint distribution of the parameters. These distributions are estimated empirically by iteratively sampling each parameter. Given the empirical joint distribution the mean and 95% confidence interval are then easily calculated. We used 5000 MCMC samples with a burn-in of 1000.

The model assumes that the residual noise is normally distributed, and this assumption was checked using the Shapiro-Wilk test. Because the original paper by Wohlfahrt and colleagues [11] reported a bimodal distribution of birth length, we used a model with two peaks at 6 and 12 month periods (bimodal model). Full details of the program (written in SAS) can be downloaded from the WHO MONICA website [25].

We combined data for males and female babies. Although males are generally heavier than females at birth, the trends over time and seasonal changes in weights and lengths were similar. Hence combining the data for this analysis is justified. We plotted the estimated trends (and 95% confidence intervals) in birth weight and length over time. We also plotted estimates of the overall seasonal pattern by time. Because the time series is long, we plotted seasonal patterns once every five years, and also for the last year (2002).

### Modelled time series data

In order to explore the assumptions of standard seasonal analyses, we first modelled time series data with a seasonal (i.e. 12 month) periodicity, but varied both the nature of the exposure-risk relationship (e.g. linear, non-linear), and the distribution of the population within the exposure range. The exposure-risk relationship is analogous to a dose-response relationship. The outcome may not always be a risk (e.g. odds of disease), but may be a continuous measure (e.g. birth weight).

If different populations are exposed to limited doses of the exposures (e.g. as might be expected at different latitudes for temperature and ultra-violet radiation-related exposures), then the resulting time series may also reflect the 'local' properties of the non-linear curve (i.e. the population maps on to a limited section of the J-shaped curve). We assumed a single symmetric sinusoidal seasonal exposure (a period of 12 months), with a peak in January. We examined a range of likely exposure-risk relationships and generated the associated time series of seasonal risk. The exposure-risk and seasonal-risk relationships were plotted. In a second analysis we varied the Population Exposure Distribution (PED) into low, medium and high groups. We assigned a number of different weights to these groups to simulate different populations.

Finally, by way of demonstration, we attempted to build a model that mimics key aspects of the Danish perinatal data. We assumed a fixed J-shaped ERR across time, and examined the impact of shifting the PED on the seasonal distribution.

## Results

### Seasonality in Danish neonatal anthropometry

The Danish Medical Birth Register included 1 573 203 neonates (765 438 females, 807 765 males). Over the entire epoch, the mean (and standard deviation) for birth weight in males and females was 3533.2 (578.9) and 3412.0 (545.8) grams respectively. For birth length, the mean (and standard deviation) for males and females was 52.2 (2.6) and 52.4 (2.5) centimetres respectively.

**Figure 1:**
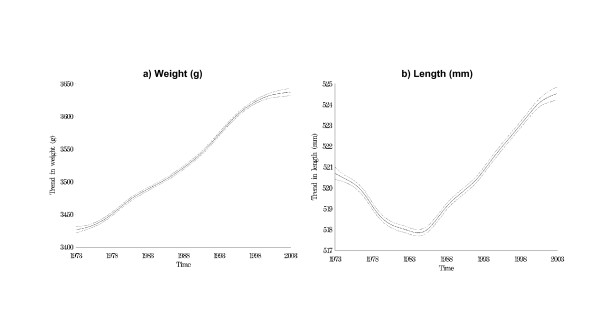
Plot of mean trend in birth weight and length and 95% confidence intervals. (Raw data).

Figures 1a and 1b show the plot of mean trend in birth weight (1a) and birth length (1b) and 95% confidence intervals based on the original data. There were no substantial sex differences in secular trends in the neonatal anthropometric measures (data not shown). Note that the long-term trend in birth length changes around 1983–4. After this date, weight and length rise over time in a similar fashion.

**Figure 2:**
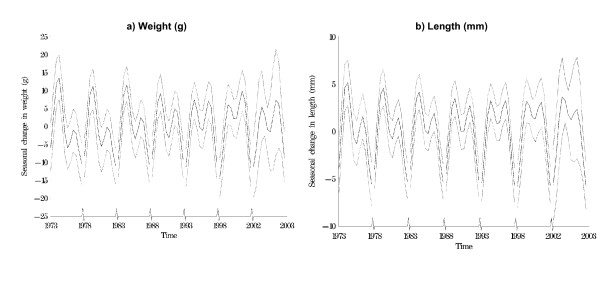
Seasonal changes in weight and length and 95% confidence intervals. Results plotted every five years and 2002. (Data derived from 'combined analysis').

Figures 2a and 2b show six five-year (and one one-year) epochs for weight and length derived from the 'combined method' previously described. When plotted in these 'bins', the changes over time in seasonal features of these measures are apparent. Note that for both anthropometric measures, the amplitude of the larger spring peak has diminished over time, while the small summer peak has increased slightly. Before 1997, the highest monthly mean birth weight was in April (spring). After 1997 the highest monthly mean birth weight was September. Before 1989 the lowest mean birth weight was always in December (early winter). After 1989, the lowest was either December or January. A similar pattern was found for birth length. The highest mean birth length was in April for every year except for 1993 and 1994 when it was September. The lowest birth length was in December or January. The findings were unchanged when males and females were examined separately (data not shown). There was a gradually decreasing peak mean birth weight in April over time. For both sexes the mean increase in birth weight in April was 13.5 grams in 1973 and 5.4 grams in 2002.

### Illustrations of the impact of non-linear ERR on seasonality

**Figure 3:**
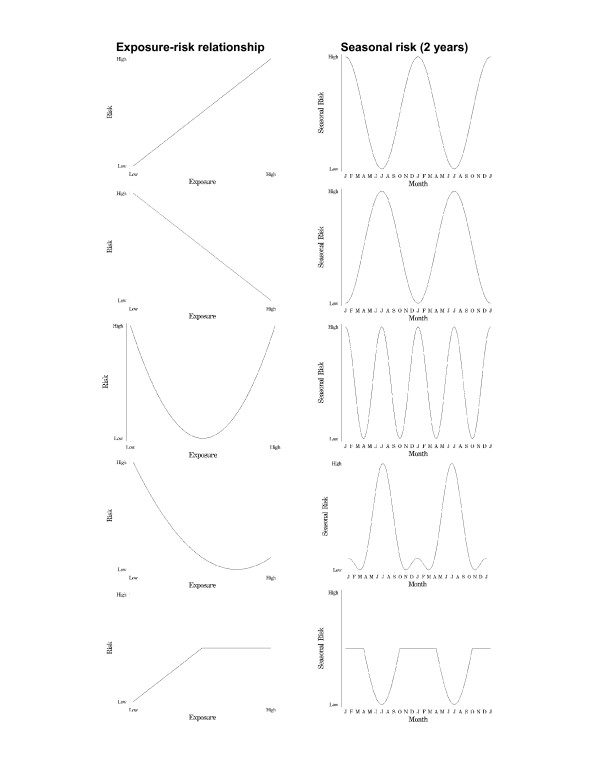
The relationship between the exposure-risk relationship and the seasonal pattern of risk (over 2 years).

Concerning the first modelling exercise, Figure 3 demonstrates the relationship between different linear and non-linear ERRs and the derived seasonal time series. A linear ERR with either a positive or negative slope led to a seasonal risk pattern with a 12 month period. There was a difference in phase of 6 months between the negative and positive linear ERR.

Changing the ERR from linear to U-shaped generated a time series with a 6 month periodicity (i.e. double the frequency of the parent 12 month periodicity). Altering the ERR to J-shaped partitioned the resulting time series in a dominant 12 month signal and a secondary 12 month signal phase-shifted by 6 months.

**Figure 4:**
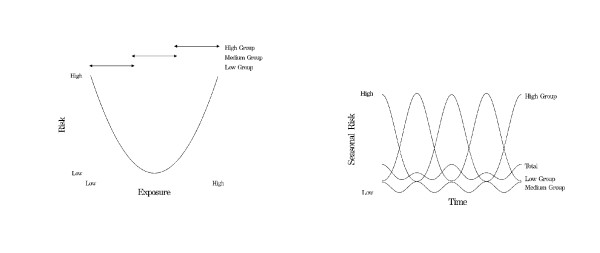
The relationship between a U-shaped exposure-risk relationship and the seasonal pattern in risk (over 2 years), after partitioning the population into three exposure groups.

The seasonal patterns in Figure 3 assumed an equal and full range of exposure in the population. In a second modelling exercise (Figure 4) we examined the effect of partitioning the population into different risk categories with a U-shaped ERR. This resulted in an unexpected pattern of two dominant 12 month signals, phase-shifted by 6 months, and a smaller signal with a 6 month periodicity. The sum of these time series produced an overall time series with an unexpectedly high risk status all year, but with a 6 month period and relatively small amplitude.

### Modelling Danish data

**Figure 5:**
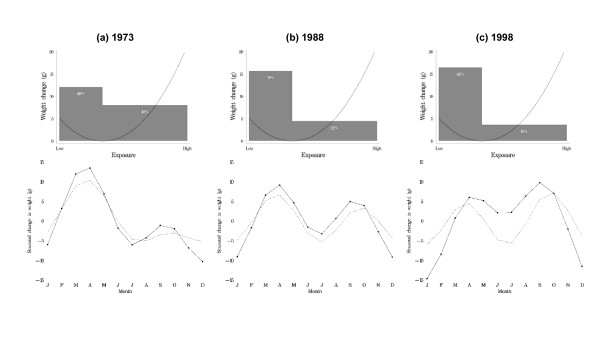
Seasonal change in Danish birth weight data (combined results for boys and girls) for three years (1973, 1988 and 1998), and fitted seasonal change based on a J-shaped exposure-risk relationship and a shifting population exposure over time. The first row of figures shows the hypothesised J-shaped exposure-risk relationship and the percent of the population in the low versus mid-high exposure groups. The second row of figures shows the estimated seasonal risk based on the observed data (solid line) and the estimated risk based on the J-shaped exposure-risk relationship (dotted line).

Finally, Figure 5 demonstrates the effect of an altered PED on seasonal fluctuations based on a J-shaped ERR. Panel 5a recreates the seasonal pattern for 1973 by allocating 60% of the population to the low range and 40% to the mid-high range. Panel 5b recreates the seasonal pattern for 1988 by allocating 78% of the population to the low range and 22% to the mid-high range. Panel 5c recreates the seasonal pattern for 1998 by allocating 82% of the population to the low range and 18% to the mid-high range.

## Discussion

In keeping with a previous study also based on the Danish population sample [11], we found that both the birth weight and birth length of Danish neonates displayed prominent seasonal fluctuations. The amplitude of these seasonal fluctuations changed over time. The features of these fluctuations are consistent with models based on J-shaped ERR and left-shifting the PED over time (i.e. the population distribution of the exposure is shifted down to low values).

In general, we note that J-shaped or U-shaped models of exposure-risk result in substantial changes in the nature of seasonally-generated time series. The findings have implications for those wishing to synthesise or understand diverse and apparently conflicting seasonal data. Time series with both 6 and 12 month periodicities may be the result of an exposure with a J-shaped exposure-risk relationship.

Furthermore, if researchers are not aware of the underlying U- or J-shaped models, different populations facing different doses of the exposure could result in conflicting results. For example, as shown in Figure 4, a population with a low exposure would be restrained within one side of the U-shaped curve. This results in a negative correlation between the variables of interest. Studies done in populations restrained to the opposite side of the U-shaped curve would find a significant positive correlation, and a time series with peak 6 months shifted from the previous example. Populations in the base of the U-shape would find a relatively small amplitude, with an even, 6 month periodicity. Altering the distribution of the population around this curve results in summed signals with unexpected properties.

The results of this study highlight that interpreting overall seasonal patterns should be done with care. The simplest interpretation requires the assumption that exposure is constant across the population. Many researchers examining seasonal patterns may not realise that they are making this assumption, or the implications if it is violated. This could lead to them wrongly concluding that two seasonal exposures are at work (e.g. a 6 and 12 month exposure).

Our analyses of Danish birth anthropometric data clearly show that the amplitudes did not remain constant over time (Figure 2). This means that the time series of birth weights and lengths are non-stationary. Hence the assumptions of most standard time series methods–such as those based on the periodogram, or those that average results over months–are violated, and would have given incorrect estimates of the seasonal pattern. The method used here was able to model the non-stationarity, and hence gave a more accurate fit to the data.

Depending on the cancellation of seasonal components (as shown in Figure 4), a study may not have enough statistical power to detect an overall seasonal change in risk. Power depends on the amplitude of the seasonal change and the amount of data (number of subjects and length of time). This could lead researchers to wrongly conclude that a health outcome is not seasonal, when in fact its seasonality is strong but depends on group.

We note with interest that our new model provides a parsimonious explanation of the interaction between latitude and seasonal fluctuations in birth weight noted in both Japan [8] and Australia [14]. Both of these studies reported bimodality (6 and 12 month periodicity). Both studies reported a prominent peak in spring and a secondary peak in autumn. Both studies reported that the amplitude of the spring peak was larger in sites closer to the equator. Researchers interested in synthesising these results may wish to examine candidate exposures that have both (a) non-linear ERRs, and (b) latitude gradients (i.e. the PED of the exposure will vary across latitudes).

With respect to seasonally-fluctuating exposures that may underlie these findings, we speculate that low prenatal vitamin D is a candidate that warrants closer inspection [4]. Firstly, there is robust evidence that vitamin D levels fluctuate over the seasons and also across latitudes [26]. Secondly, a nonlinear exposure-risk relationship between maternal vitamin D levels and neonatal anthropometry has been found [22]. The high prevalence of hypovitaminosis D in pregnant woman and the potential adverse health outcomes for the fetus has been the focus of recent editorial comments [27,28]. For example, low maternal vitamin D levels have been associated with altered birth anthropometry, with some studies finding an association between vitamin D deficiency and larger birth weight and length [29], while others have shown an association between lower maternal vitamin D levels and shorter gestation and smaller knee-heel length (a component of birth length) [22]. Future studies would ideally examine the relationship between maternal vitamin D status and neonatal anthropometry across time (e.g. in different seasons) and in a range of sites (e.g. high and low latitude).

Apart from neonatal anthropometry, J-shaped ERRs have also been observed between temperature and cardiovascular disease [30,31], with large increases in risk associated with cold-temperatures and smaller increases with hot temperatures. A J-shaped ERR may provide a parsimonious explanation for the observed 6 and 12 month bi-modal peaks in seasonal cardiovascular disease risk [13,32].

With respect to the Danish data, our findings are in keeping with other studies [11,33]. Mean birth weight rose in a steady linear fashion over the period of observation (1973–2002). Curiously, mean birth length appeared to decline from 1973 to approximately 1985, and then rose steadily in a parallel fashion to birth weight. Assuming that the population mix remained stable over this period (i.e. that the broad genetic 'mix' of the Danish population was essentially stable, which is a fair assumption since both the child and its parents were born in Denmark), the change in secular trend in birth length suggests that factors that impact on fetal growth changed over this time. The mechanisms underlying this change remain unclear. There is evidence showing that the estimation of gestational age improved over recent decades [34], however this can not readily explain the nature of the current findings.

The time series in the modelling exercises shown above were generated without any noise and thus show ideal shapes. In real-life situations measurement and random error will lead to observed time series that are noisier than those shown here. Methods to extract the seasonal component after removing noise and trend should be used [13]. We have limited our modelling by only investigating models based on biologically plausible assumptions – many exposures have J- and U-shaped ERRs. Furthermore, the PED changes we used in the models are relatively modest and within the range of many latitude-associated exposures (e.g. vitamin D, temperature). Other combinations of exposure-risk relationships (e.g. S- or V-shaped) and population exposure distributions may also give good fits to the observed seasonal pattern. The range of possible ERRs and PEDs is very large, and it would require a lot of computer power to search through all possible models. Such a search would also be data-driven rather than hypothesis-driven. Nevertheless, it is still important to note that the models of seasonal change shown here are just one possible fit to the data. Modelling can not replace a thorough understanding of the biological properties of candidate exposures. As with all epidemiology, analytic methods such as the choice of linear versus non-linear ERRs should be built on the best-available biology and experimental data. Conversely, signals that emerge from observational epidemiology can also help guide more focussed biological research (e.g. dose-response studies in animal models in order to refine the properties of the ERR).

## Conclusion

Epidemiology continues to explore the role of seasonality in a surprisingly wide range of health outcomes [35]. More sophisticated statistical methods and more realistic models of the exposure-risk relationship should assist the research community to 'decipher' the seasonally-fluctuating signal from complex time series data.

We propose that non-linear Exposure-Risk Relationships provide parsimonious models of time data series with both 6 and 12 month periodicities. Changing the Population Exposure Distribution in these models leads to surprising and informative changes in the overall seasonal risk. We believe that non-linear models can provide conceptually elegant solutions to a range of seasonally-fluctuating health outcomes.

## List of abbreviations

ERR = Exposure-Risk Relationship

PED = Population Exposure Distribution

## Competing interests

The author(s) declare that they have no competing interests.

## Authors' contributions

JM and AB designed the study. AB performed the analyses. PBM and CBP provided the birth weight data. DE and TB contributed to the interpretation of the data. All authors contributed to the interpretation of the data and writing up, and all authors read and approved the final document.

## Pre-publication history

The pre-publication history for this paper can be accessed here:


